# Preliminary Phytochemical Screening and Biological Activities of *Bulbine abyssinica* Used in the Folk Medicine in the Eastern Cape Province, South Africa

**DOI:** 10.1155/2015/617607

**Published:** 2015-10-12

**Authors:** Cromwell Mwiti Kibiti, Anthony Jide Afolayan

**Affiliations:** Medicinal Plants and Economic Development (MPED) Research Centre, Department of Botany, University of Fort Hare, Alice 5700, South Africa

## Abstract

*Bulbine abyssinica* A. Rich. is used in traditional medicine to treat rheumatism, dysentery, bilharzia, cracked lips, back pain, infertility, diabetes mellitus, and gastrointestinal, vaginal, and bladder infections. Therefore, preliminary phytochemical screening, antioxidant, anti-inflammatory, and antibacterial properties of the whole plant (acetone and aqueous extracts) were determined using standard procedures. The *in vitro* antioxidant model assays revealed that the plant possesses free radical scavenging potential varying with free radical species. The species showed significant protein denaturation inhibitory activity with good protection against erythrocyte membrane lysis indicating anti-inflammatory potential. The results also showed that the species was active against the growth of all the selected eight diabetic status opportunistic bacteria except one. Moreover, the species is characterized by appreciable amounts of total phenols, flavonoids, flavanols, proanthocyanidins, and alkaloids. Traces amounts of saponins and tannins were also observed. Amongst the identified phytochemicals present, empirical searches identified them being antioxidant, anti-inflammatory, and antimicrobial agents. The identification of these phytochemical constituents with their known pharmacological properties indicates that this plant is a good source of the free radical scavenging, anti-inflammatory, and antimicrobial agents. These findings also account for the multipharmacological use of *B. abyssinica* in fork medicine.

## 1. Introduction

Many diseases are caused by oxidative stress that results from imbalance between the formation and neutralization of free radicals [1]. Oxidative stress initiated by reactive oxygen species (ROS) such as superoxide anions, hydrogen peroxide, hydroxyl, nitric oxide, and peroxynitrite damages cellular macromolecules such as DNA, proteins, and lipids [2]. Among the effects, lipid peroxidation initiates inflammation processes. Therefore, inflammation is intertwined to oxidative stress [3].

The mechanism of inflammation is also ascribed with the release of ROS, stimulating the release of inflammation factors such as cytokines which activates release of neutrophils and macrophages. During inflammatory pathogenesis, there is an excessive activation of phagocytes, production of free radicals which increase vascular permeability, protein denaturation, and membrane alteration [4]. Thus, free radicals are vital mediators that provoke/sustain inflammatory processes and consequently, their neutralization by antioxidants can attenuate inflammation [4]. This cellular oxidative damage may result in diseases including diabetes mellitus, atherosclerosis, myocardial infarction, and neurodegenerative diseases [5]. Microbial invasion during diabetes mellitus status and in other disease conditions is attributed to the host having susceptible damaged cells due to inflammation [5].

Diabetic patients are prone to develop bacterial and fungal infections [6]. The common microbes implicated in these infections include* Streptococcus pneumonia*,* Escherichia coli*,* Staphylococcus aureus*,* Staphylococcus epidermidis*,* Pseudomonas aeruginosa*, and* Candida albicans* [7].* Shigella flexneri*,* Proteus vulgaris*,* Klebsiella pneumoniae*, and* Enterococcus faecalis* lead to diabetes foot ulcer [8–11]. The bacteria associated with gastrointestinal and urinary tract infections include* Klebsiella pneumoniae*,* Streptococcus pyogenes*, and* Serratia marcescens* [12–14]. Therefore, plant remedies are known to attenuate these infections through acting as antimicrobial agents or by reducing/neutralizing ROS generated during pathophysiology of these diseases and/or by reducing inflammation status [15].

Human cells have an array of protective mechanisms in prevention of the production of free radicals and attenuation of oxidative damage [16]. These mechanisms include release of enzymatic and nonenzymatic antioxidants such as superoxide dismutase, catalase, glutathione reductase, glutathione peroxidase, ascorbic acid, and tocopherol [16]. The protective roles of these enzymes may be disrupted as a result of various pathological processes, thereby causing damage to the cells. Therefore, the cells also offer protection against inflammation via inhibiting protein denaturation agents and protection against membrane lysis [17]. Synthetic antioxidants (such as butylated hydroxyanisole and butylated hydroxytoluene) and nonsteroidal inflammatory drugs (such as diclofenac sodium and aspirin) are commercially available and currently used [17]. However, these drugs have side effects; hence this has led to seeking alternative medicine from plant remedies [17].

Medicinal plants play important roles as source of antioxidant, inflammatory, and antibacterial agents. These bioactivities are mainly due to the presence of phenolic compounds [18]. Among these plants is the* Bulbine abyssinica* A. Rich. (Asphodelaceae).* Bulbine abyssinica* is a succulent perennial herb with rhizomatous base which grows in small clusters. The plant is a water-wise plant with both flowers and fruits having yellow and black colors. The roots are many, slender or swollen.* B. abyssinica* occurs from the Eastern Cape, through KwaZulu-Natal, Swaziland, and Lesotho, and further north to Ethiopia [19].

*B. abyssinica* is used in South Africa fork medicine to treat rheumatism, dysentery, bilharzia, cracked lips, infertility, back pain, and gastrointestinal, vaginal, and bladder infections [20]. A decoction prepared from the whole plant is used in the management of diabetes mellitus [21].

Studies have shown that the stems and roots of* Bulbine* species contain anthraquinones that possess antibacterial properties [22]. Anthraquinones, phenylanthraquinones, and isofuranonaphthoquinones have also been isolated from the roots, leaves, and fruits of this plant [22]. The phenylanthraquinone isolated from the roots has* in vitro* antiplasmodial activity and no cytotoxic effects on mammalian cell lines [22].

Though some of the* B. abyssinica*'s chemical compounds and bioactivities from some plant's parts have been elucidated, the phytochemical constituents, antioxidant, anti-inflammatory, and antibacterial properties remain obscure. Therefore, the aim of this study was to quantify the presence of some phytochemical constituents and evaluate these pharmacological activities of acetone and aqueous whole plant extracts of* B. abyssinica* used in the treatment of diabetes mellitus and associated infections using standard procedures and relate our findings to their folklore uses.

## 2. Materials and Methods

### 2.1. Plant Collection and Preparation

The whole plant of* B. abyssinica* including the leaves, flowers, stems, and roots was collected from lower Ncera location in Nkonkobe Municipality of the Eastern Cape Province, South Africa. The voucher specimen (KibMed 2014/01) was deposited in Giffen's herbarium, University of Fort Hare, South Africa, for authentication.

### 2.2. Extraction Methods

The plant samples were air-dried, ground to homogeneous powder, and extracted using acetone and water. For acetone extraction, the solvent and ground samples were mixed on a shaker for 48 h, while aqueous extraction was done by boiling the samples in distilled water for 30 min and let to cool. These extracts were then filtered. The filtrate obtained with water extraction was frozen at −40°C and freeze-dried for 48 h using a freeze dryer (Vir Tis benchtop K, Vir Tis Co., Gardiner, NY). The acetone extracts were concentrated to dryness under reduced pressure at 57°C using a rotary evaporator (Strike 202 Steroglass, Italy). The resulting extracts were reconstituted with their respective solvents to give the desired concentrations used in the study.

### 2.3. Chemicals and Reagents Used

All the chemicals used were of analytical grade and were purchased from Merck and Sigma-Aldrich, Gauteng, South Africa.

### 2.4. Phytochemical Analysis

#### 2.4.1. Determination of Total Phenols

The total phenolic content in the extracts was determined by the modified Folin-Ciocalteu method [23]. An aliquot of 0.5 mL of each plant extract (1 mg/mL) was mixed with 5 mL of Folin-Ciocalteu reagent which was previously diluted with distilled water (1 : 10 v/v) and 4 mL (75 g/L) of sodium carbonate. The mixtures were vortexed for 15 sec and allowed to stand for 30 min at 40°C to develop color. Absorbance was then read at 765 nm using the AJI-C03 UV-Vis spectrophotometer. The results were expressed as mg/g tannic acid equivalent using the equation based on the calibration curve:

(1) Y=0.1216x;R2=0.9365,

where *x* is the absorbance and *Y* is the tannic acid equivalent.

#### 2.4.2. Determination of Total Flavonoids

The flavonoid content was determined by the method used by Oyedemi et al. [24]. Briefly, 0.5 mL of 2% AlCl_3_ was prepared in ethanol. This was then added to 0.5 mL of the extracts. The mixture was allowed to stand for 60 min at room temperature and the absorbance was read at 420 nm. The extracts were evaluated at a final concentration of 0.1 mg/mL. The results were calculated as quercetin equivalent (mg/g) using the equation based on the calibration curve:

(2) Y=0.0255x;R2=0.9812,

where *x* is the absorbance and *Y* is the quercetin equivalent.

#### 2.4.3. Determination of Total Flavonols

The flavonol content was determined based on the method used by Oyedemi et al. [24]. Briefly, 2 mL of each plant extract was mixed with 2 mL of AlCl_3_ prepared in ethanol. Then 3 mL of sodium acetate solution (50 g/L) was added. The mixture was incubated at 20°C for 2.5 h. Absorbance was measured at 440 nm. The total flavonol content was calculated as quercetin (mg/g) using the following equation based on the calibration curve:

(3) Y=0.0255x;R2=0.9812,

where *x* is the absorbance and *Y* is the quercetin equivalent.

#### 2.4.4. Determination of Proanthocyanidin

The total proanthocyanidin was determined using the method described by Oyedemi et al. [24]. A volume of 0.5 mL of the extract solution was mixed with 3 mL of 4% vanillin-methanol solution and 1.5 mL HCL. The resulting mixture was vortexed and allowed to stand for 15 min at room temperature and absorbance read at 500 nm. Total proanthocyanidin content was expressed as catechin equivalents (mg/g) using the calibration curve equation:

(4) Y=0.5825x;R2=0.9277,

where *x* is the absorbance and *Y* is the catechin equivalent.

#### 2.4.5. Determination of Tannins

Tannin determination was done according to the procedure of Mbaebie et al. [25] with some modifications. 0.2 g of plant extract was added to 20 mL of 50% methanol. This was mixed thoroughly and placed in a water bath at 80°C for 60 min. The extract was filtered into a 100 mL volumetric flask; 20 mL of distilled water was added, followed by 2.5 mL of Folin-Ciocalteu reagent and 10 mL of 17% Na_2_CO_3_. This was thoroughly mixed together and made up to 100 mL using distilled water. The mixture was allowed to stand for 20 min until the bluish-green color developed. The different tannic acid standard solutions concentrations used ranged from 0 to 10 ppm. The absorbance of the tannic acid standard solutions and plant extracts were measured after color development at 760 nm using the AJI-C03 UV-Vis spectrophotometer. The results were expressed as mg/g of tannic acid equivalent using the calibration curve:

(5) Y=0.0763x;R2=0.9644,

where *x* is the absorbance and *Y* is tannic acid equivalent.

#### 2.4.6. Determination of Alkaloids

The alkaloid content was determined according to the method of Omoruyi et al. [26]. Briefly, 5 g of plant extract was mixed with 200 mL of 10% acetic acid in ethanol. The mixture was covered and allowed to stand for 4 h. This was filtered and the filtrate was concentrated on a water bath to one-fourth of its original volume. Concentrated ammonium hydroxide was added in drops to the extract until precipitation was completed. The whole solution was allowed to settle and the collected precipitates were washed with dilute ammonium hydroxide and then filtered. The residue collected was dried and weighed. The alkaloid content was determined using the following formula:

(6) $$\begin{array}{c} \%\;\;alkaloid=\frac{\text{final weight of sample}}{\text{initial weight of extract}}\times 100. \end{array}$$

#### 2.4.7. Determination of Saponins

The saponin content in the plant extracts was determined using the method of Omoruyi et al. [26]. Briefly, 20 g of the plant extract was mixed with 200 mL of 20% ethanol in a shaker for 30 min and heated in a water bath at 55°C for 4 h with continuous stirring. The mixture was filtered and the residue was reextracted with another 200 mL of 20% ethanol. The combined extracts were reduced to 40 mL over the water bath at 90°C. The concentrated solution obtained was then transferred into a 250 mL separating funnel and extracted twice using 20 mL diethyl ether. The ether layer was discarded, while the aqueous layer was retained and 60 mL* n*-butanol was added. The* n*-butanol extracts were washed twice with 10 mL of 5% sodium chloride. The remaining solution was heated in a water bath to evaporate and the samples were oven dried at 40°C to a constant weight. The percentage saponin content was calculated using the following formula:

(7) $$\begin{array}{c} \%\;\;saponin=\frac{\text{final weight of sample}}{\text{initial weight of sample}}\times 100. \end{array}$$

### 2.5. *In Vitro* Antioxidant Analysis

#### 2.5.1. Antioxidant Assay

The antioxidant activities of the acetone and aqueous extracts of* B. abyssinica* whole plant were determined using DPPH, nitric oxide, reducing power, hydrogen peroxide, ABTS, and lipid peroxidation inhibitory assays.

#### 2.5.2. Ferric Reducing Power Assay

The reducing power of the plant extracts was determined by the method of Mamta et al. [27] with slight modifications. Briefly, different concentrations (0.025–0.5 mg/mL) of extracts (0.5 mL) were mixed with 0.5 mL 0.2 M phosphate buffer (pH 6.6) and 0.5 mL 0.1% potassium hexacyanoferrate, followed by incubation at 50°C in a water bath for 20 min. After incubation, 0.5 mL 10% trichloroacetic acid was added to terminate the reaction. The upper portion of the solution (1 mL) was mixed with 1 mL of distilled water and 0.1 mL 0.01% FeCl_3_ solution added. The reaction mixture was left for 10 min at room temperature and the absorbance measured at 700 nm against the appropriate blank solution. A higher absorbance of the reaction mixture indicated greater reducing power.

#### 2.5.3. DPPH Radical Scavenging Activity Assay

The method of Mamta et al. [27] was used for the determination of DPPH free radical scavenging activity. Briefly, a solution of 0.135 mM DPPH radical in methanol was prepared. 1 mL of this solution was mixed with 1.0 mL of each extract (0.025 to 0.5 mg/mL) and standard drugs (BHT and ascorbic acid) (0.025 to 0.5 mg/mL). The reaction mixture was then vortexed thoroughly and left in the dark at room temperature for 30 min. The absorbance of the mixture was measured spectrophotometrically at 517 nm. The actual decrease in absorbance was measured against that of the control. The scavenging ability of the plant extract was then calculated using the following equation:

(8) DPPH Scavenging activity %=Abs control−Abs sampleAbs control×100,

where Abs control is the absorbance of DPPH + methanol and Abs sample is the absorbance of DPPH radical + sample (sample or standard).

#### 2.5.4. Nitric Oxide Scavenging Activity Assay

The modified method described by Oyedemi et al. [24] was used to determine the nitric oxide radical scavenging activity. A volume of 2 mL of 10 mM of sodium nitroprusside prepared in 0.5 mM phosphate buffer saline (pH 7.4) was mixed with 0.5 mL of plant extracts, gallic acid, and BHT individually to make different concentrations from 0.025 to 0.5 mg/mL. The mixture was incubated at 25°C for 150 min. Then, 0.5 mL of incubation solution was then mixed with 0.5 mL of Griess reagent: 1.0 mL sulfanilic acid reagent (0.33% prepared in 20% glacial acetic acid at room temperature for 5 min with 1 mL of naphthylethylenediamine dichloride (0.1% w/v)). The mixture was incubated at room temperature for 30 min and absorbance taken at 540 nm. The amount of nitric oxide radicals inhibited by the plant fraction/standard drugs was calculated using the following equation:

(9) NO radical scavenging activity %=Abs control−Abs sampleAbs control×100,

where Abs control is the absorbance of NO radicals + methanol and Abs sample is the absorbance of NO radical + extract or standard.

#### 2.5.5. Lipid Peroxidation Scavenging Activity Assay

The inhibition of lipid peroxidation in the rat liver homogenate was determined using a modified thiobarbituric acid reactive species (TBARS) assay as described by Murugan and Parimelazhagan [3]. The liver homogenate (0.5 mL, 10% in distilled water, v/v) and 0.1 mL of each plant extract were mixed separately in a test tube and the volume was made up to 1 mL by adding distilled water. Then, 0.05 mL FeSO_4_ (0.07 M) was added to the above mixture and incubated for 30 min to induce lipid peroxidation. Thereafter, 1.5 mL of 20% acetic acid and 1.5 mL of 0.8% TBA (w/v) in 1.1% sodium dodecyl sulfate and 0.05 mL 20% TCA were added, vortexed, and then heated in a boiling water bath for 60 min. After cooling, 5.0 mL of butanol was added to each tube and centrifuged at 3000 rpm for 10 min. The absorbance of the organic upper layer was measured at 532 nm. For the blank, 0.1 mL of distilled water was used in place of the extract. Inhibition (%) of lipid peroxidation was calculated using the following equation:

(10) %  Inhibition=Control OD−Sample ODControl OD×100.

#### 2.5.6. Hydrogen Peroxide (H_2_O_2_) Radical Scavenging Activity Assay

The H_2_O_2_ inhibition activity of the extracts was assessed by the method of Gülçin et al. [28]. Briefly, a solution of 4 mM H_2_O_2_ was prepared in phosphate buffer (0.1 M; pH 7.4) and incubated for 10 min. One milliliter of each plant extract (0.025 to 0.5 mg/mL) was added to a 0.6 mL of hydrogen peroxide solution. The absorbance of the hydrogen peroxide at 230 nm was determined after 10 min against a blank solution containing phosphate buffer solution without hydrogen peroxide. The positive controls used were BHT (0.025 to 0.5 mg/mL) and vitamin C (0.025 to 0.5 mg/mL). The percentage scavenging of hydrogen peroxide of samples was calculated using the following formula:

(11) H2O2 inhibition capacity %=1−H2O2 cons. of sampleH2O2 cons. of blank×100.

#### 2.5.7. 2,2′-Azino-bis(3-ethylbenzthiazoline-6-sulfonic acid) (ABTS) Radical Scavenging Assay

The method described by Gülçin et al. [28] was adopted for the determination of ABTS scavenging activity. Briefly, the stock solutions including 7 mM ABTS solution and 2.4 mM potassium persulfate solution were prepared. The working solution was then prepared by mixing the two stock solutions in equal proportions and allowing them to react for 12 h at room temperature in the dark. The solution was then diluted by mixing 1 mL ABTS^+^ solution with 60 mL of methanol to obtain an absorbance of 0.708 ± 0.001 units at 734 nm using the spectrophotometer. The plant extracts (1 mL) and their controls were allowed to react with 1 mL of the ABTS^+^ solution and the absorbance was taken at 734 nm after 7 min using the spectrophotometer. The ABTS^+^ scavenging capacity of the extract was then compared with that of the standards. The percentage inhibition was then calculated as follows:

(12) $$\begin{array}{c} \text{Inhibition}\;\;\%=\left[\;\frac{\left(A\text{ blank}-A\text{ sample}\right)}{A\text{ blank}}\;\right]\times 100, \end{array}$$

where *A* blank is the absorbance of ABTS radical + methanol used as control and *A* sample is the absorbance of ABTS radical + sample extract/standard.

All antioxidant assays were done in triplicate. The activity was expressed as 50% inhibitory concentration (IC_50_). The lower the IC_50_ value, the higher the antioxidant activity.

### 2.6. *In Vitro* Anti-Inflammatory Activity

#### 2.6.1. Protein Denaturation Method

The protein denaturation assay was determined using a modified method as described by Murugan and Parimelazhagan [3]. Briefly, the reaction mixture (0.5 mL; pH 6.3) consisted of 0.45 mL of bovine serum albumin (5% aqueous solution) and 0.05 mL of distilled water. The pH was adjusted to 6.3 using a small amount of 1 N HCL. 1 mL of acetone or aqueous extract with final concentrations of (0.1 to 0.5 mg/mL) was added to the reaction mixture. These were incubated at 37°C for 30 min and then heated at 57°C for 5 min. After cooling the samples, 2.5 mL of phosphate buffer solution (pH 6.4) was added. Turbidity was measured spectrophotometrically at 660 nm. For the negative control, 0.05 mL of distilled water and 0.45 mL of bovine serum albumin were used. Diclofenac sodium with the final concentration of 100, 200, 300, 400, and 500 *μ*g/mL was used as reference drug. The percentage inhibition of protein denaturation was calculated by using the following formula:

(13) Percentage Inhibition %=Abs control−Abs sampleAbs control×100.

### 2.7. Membrane Stabilizing Activity

#### 2.7.1. Hypotonic Solution-Induced Rat Erythrocyte Haemolysis

The rat erythrocyte cells were prepared using the method described by Majumder et al. [29]. Briefly, the whole blood was obtained with heparinized syringes from a rat through cardiac puncture. The blood was washed three times with isotonic buffered solution (154 mM NaCl) in 10 mM sodium phosphate buffer (pH 7.4). The blood was centrifuged each time for 10 min at 13,000 rpm [29].

Membrane stabilizing activity of the extracts was assessed using hypotonic solution-induced rat erythrocyte haemolysis method as described by Majumder et al. [29]. Briefly, the test sample consisted of stock erythrocyte (RBC) suspension (0.5 mL) mixed with 5 mL of hypotonic solution (50 mM NaCl) in 10 mM sodium phosphate buffered saline (pH 7.4) containing the plant extract or standard drug at concentrations ranging from 0.1 to 1 mg/mL. The control sample consisted of 0.5 mL of RBC mixed with hypotonic-buffered saline solution alone. The mixtures were incubated for 10 min at room temperature and centrifuged for 10 min at 13,000 rpm. The absorbance of the supernatant was measured at 540 nm. The percentage inhibition of haemolysis or membrane stabilization was calculated as follows:

(14) $$\begin{array}{c} \%\;\;\text{Inhibition of haemolysis}=100\times \left[\;OD1-\frac{OD2}{OD1}\;\right], \end{array}$$

where OD1 is the optical density of hypotonic-buffered saline solution alone and OD2 is the optical density of test sample in hypotonic solution.

### 2.8. Antibacterial Analysis

#### 2.8.1. Microorganisms and Media

The bacteria used in this study were chosen primarily on the basis of their importance as opportunistic pathogens of humans with diabetes mellitus [8–14]. These bacterial strains include* Shigella flexneri* KZN,* Proteus vulgaris*,* Klebsiella pneumonia* ATCC 4352,* Staphylococcus aureus*,* Enterococcus* faecalis ATCC 29212,* Streptococcus pyogenes*,* Pseudomonas aeruginosa* ATCC 19582, and* Serratia marcescens* ATCC 9986. The test organisms were obtained from the Department of Biochemistry and Microbiology, University of Fort Hare, South Africa.

Both Mueller-Hinton dextrose agar (MDA) and Mueller-Hinton dextrose broth (MDB) were prepared according to the manufacturer's instructions. The nutrient agar was suspended in demineralized water, boiled while stirring until completely dissolved. It was autoclaved at 121°C for 15 min. The bacteria were maintained at 4°C on MDA plates, and the inoculums for the assays were prepared by diluting scraped cell mass in 0.85% sodium chloride solution, adjusted to 0.5 McFarland standards, and confirmed by spectrophotometric reading at 580 nm [30]. The cell suspensions were finally diluted 1 : 100 in broth to give an approximate inoculum of 10^4^ CFU mL^−1^ as compared with McFarland standard for use in the assays [30].

#### 2.8.2. Antibacterial Susceptibility Assays

Agar diffusion and microdilution methods were used to determine the antibacterial activities of the plant's extracts against the opportunistic bacteria.

### 2.9. *In Vitro* Antibacterial Susceptibility Test

The agar well diffusion technique was employed as described by Otang et al. [30] and Prabuseenivasan et al. [13] with some modifications to determine the antibacterial susceptibility test. Briefly, 100 *μ*L of 0.5 McFarland solutions of bacterial strain cultures in 0.85% sterile distilled water (SDW) was placed over the surface of an agar plate and spread using a sterile inoculation loop. Four wells were cut in each agar plate with a cooled, flamed cork borer of 5 mm diameter, and the agar plugs removed with a sterile needle. 50 *μ*L of the positive control drug Amoxicillin (0.0125 mg/mL) and 50 *μ*L of the acetone extract at concentration of 50 mg/mL were added in the first and second wells, respectively. In the third and fourth wells, 50 *μ*L of the aqueous extract (50 mg/mL) and nutrient broth (negative control) were added, respectively. The culture plates were then incubated at 37°C, and the results were observed after 24 h. The clear zone around each well was measured in mm, indicating the activity of the plant extract against the bacterial organisms.

### 2.10. Minimum Inhibitory Concentration (MIC) Assay

The broth microdilution method using 96-well microtiter plates was employed to determine the minimum inhibitory concentration (MIC) of the plant extracts [30]. Briefly, 120 *μ*L of SDW was added into each well of the first (A) and last (H) rows and also into all the wells of the last column. Then, 120 *μ*L of nutrient broth (NB) was added into each well of the second row (B). 150 *μ*L of NB was then added into the remaining wells of the first column and 100 *μ*L into the rest of the wells from the second column rightward. Fifty microliters of the plant extract was then added into the third well of the first column while 50 *μ*L of the positive (Amoxicillin) and negative control (SDW) was separately added into the remaining wells of the first column. A twofold serial dilution was done by mixing the contents in each well of the first column (starting from the third row) and transferring 100 *μ*L into the second well of the same row and the procedure was repeated up to the 11th well of the same row and the last 100 *μ*L from the 11th well was discarded. Various concentrations of the plant extracts ranging from 5 mg/mL to 0.005 mg/mL were prepared in the wells, following the twofold dilution method.

Thereafter, 20 *μ*L of 0.5 McFarland bacteria suspensions was inoculated into the wells except those which contained SDW. The growth of the bacteria was measured by determining the absorbance at 620 nm with an automatic ELISA microplate reader (SynergyMx BiotekR, USA) before and after incubation. The plates were incubated at 37°C for 24 h. The MIC was defined and recorded as the lowest concentration of the test antibacterial agent that had inhibition on 50% bacterial growth. This was determined by assessing growth by calculating the difference in absorbance between the test wells and the control wells that had the broth and antimicrobial agent alone without the test bacteria [30].

## 3. Results

### 3.1. Phytochemical Content

**Figure 1:**
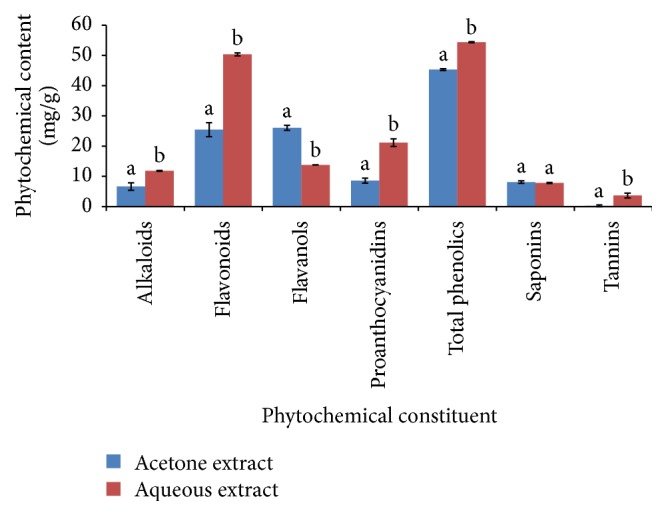
Phytochemical constituents in the acetone and aqueous extracts of* B. abyssinica*. Bar graphs with different letter superscript within the same constituent are significantly different (*P* < 0.05).

The results showed the presence of some of the assessed phytochemical constituents in varying amounts in both extracts. The aqueous extract showed significantly higher amounts of all the phytochemical constituents present than the acetone extract except total flavanols and saponin contents (*P* < 0.05) (Figure 1).

### 3.2. Antioxidant Activities of the Extracts

**Figure 2:**
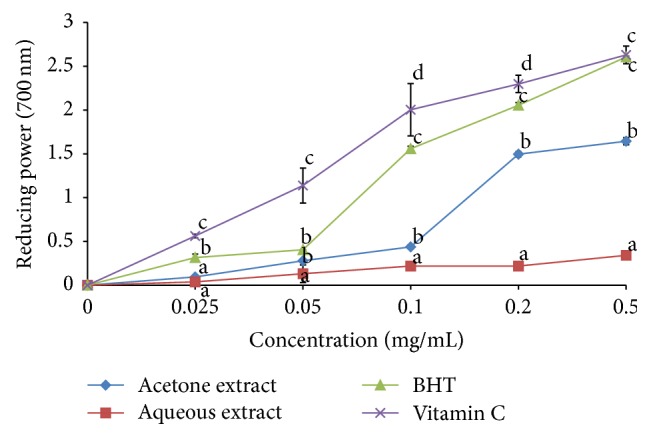
Reducing power of the acetone and aqueous extracts of* Bulbine abyssinica*. Line points with different letter superscript within the same concentration are significantly different (*P* < 0.05).

The acetone and aqueous extracts showed remarkable percentage inhibition activity in all the free radical scavenging* in vitro* models used in the present study. The antioxidant potentials of the plant extracts were estimated from their ability to reduce Fe^3+^ to Fe^2+^ which was concentration-dependent of the extracts/reference standard (Figure 2). The trend of the reducing potential of both the extracts was significantly lower than that of BHT followed by vitamin C except at 0.05 mg/mL in which acetone extract inhibitory potential was significantly similar to BHT (*P* < 0.05) (Figure 2). The IC_50_ values show that the aqueous extract had the highest Fe reducing potential compared to acetone extract but less activity when compared to the two standards (Table 1).

**Figure 3:**
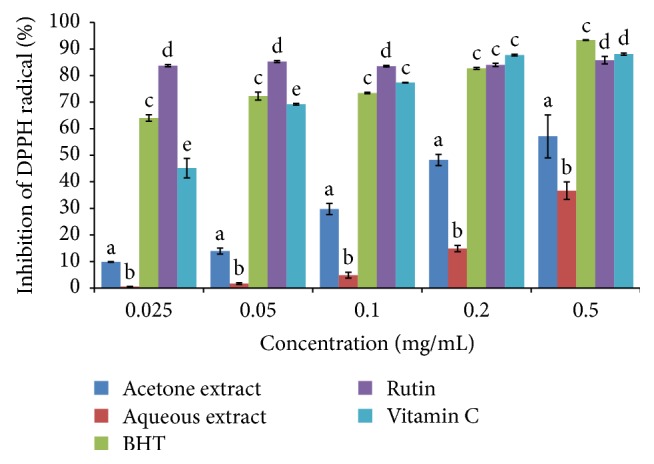
DPPH radical scavenging activity of the acetone and aqueous extracts of* B. abyssinica*. Bar graphs with different letter superscript within the same concentration are significantly different (*P* < 0.05).

The results from a series of concentration ranging from 0.025 to 0.5 mg/mL were used to determine the concentration required to attain 50% DPPH radical scavenging effect (IC_50_). A lower IC_50_ value indicates higher scavenging activity [24]. The IC_50_ values of the tested extracts/standards were in the following order: vitamin C < acetone extract < BHT < rutin ≤ aqueous extract (Table 1). Both extracts showed scavenging activity in a dose-dependent manner (*P* < 0.05) (Figure 3).

**Figure 4:**
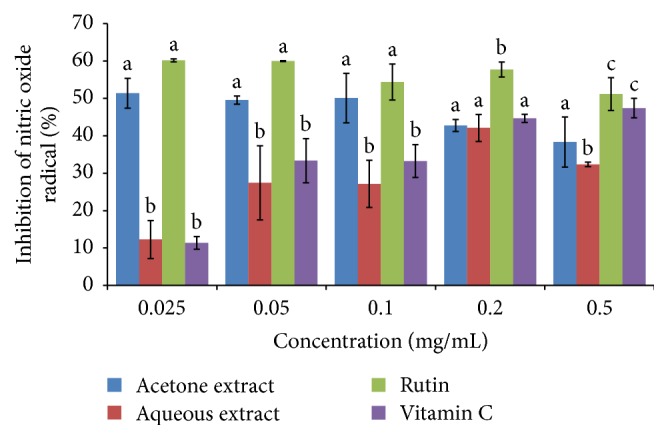
Nitric oxide radical scavenging activity of the acetone and aqueous extracts of* B. abyssinica*. Bar graphs with different letter superscript within the same concentration are significantly different (*P* < 0.05).

The nitric oxide radical scavenging activity of the plant species was revealed as the % inhibition of nitric oxide (Figure 4). The scavenging activities were concentration-dependent. The IC_50_ value determination indicates that the scavenging activity was significantly higher with vitamin C, followed by aqueous extract and then rutin and acetone (*P* < 0.05) (Figure 4) (Table 1).

**Figure 5:**
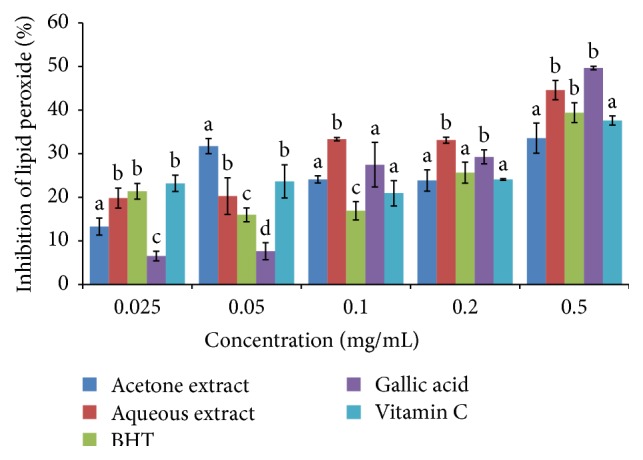
Lipid peroxidation scavenging activity of the acetone and aqueous extracts of* B. abyssinica*. Bar graphs with different letter superscript within the same concentration are significantly different (*P* < 0.05).

The scavenging of lipid peroxides by* B. abyssinica* extracts, BHT, gallic acid, and vitamin C is presented in Figure 5. The % inhibition of lipid peroxides by the extract and standards was recorded in significantly increasing order: vitamin C > BHT > gallic acid > aqueous extract > acetone extract (*P* < 0.05). The IC_50_ value indicates that acetone extract had the highest inhibitory potential followed by aqueous extract, rutin, BHT, and then vitamin C with the lowest activity (Table 1).

**Figure 6:**
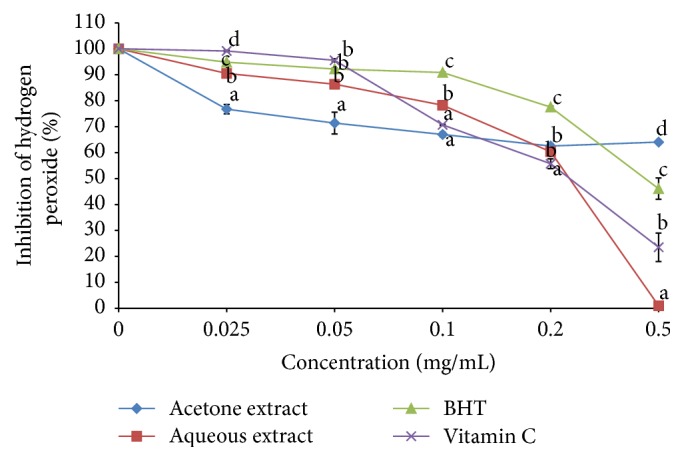
Hydrogen peroxide radical scavenging activity of the acetone and aqueous extracts of* B. abyssinica*. Line points with different letter superscript within the same concentration are significantly different (*P* < 0.05).

The IC_50_ values for H_2_O_2_ scavenging activity for acetone extract, aqueous extract, BHT, and vitamin C were 0.04, 0.24, 0.25, and 0.22 mg/mL, respectively (Table 1). The H_2_O_2_ scavenging activity of both plant species and standards decreased with increasing concentration (Figure 6). The acetone extract showed significantly higher scavenging potential than aqueous extract in all concentrations, except at 0.05 mg/mL. The scavenging activity of both plant extracts was significantly higher than those of the standards (*P* < 0.05) (Figure 6).

**Figure 7:**
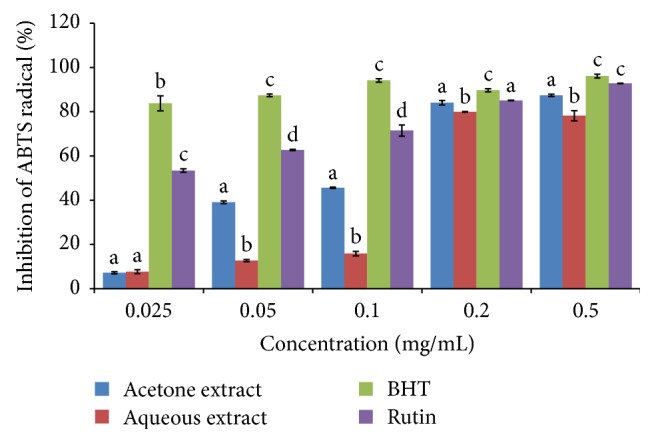
ABTS radical scavenging activity of the acetone and aqueous extracts of* B. abyssinica*. Bar graphs with different letter superscript within the same concentration are significantly different (*P* < 0.05).

The percentage inhibition of ABTS radical by the species is shown in Figure 7. The % inhibition of ABTS by the plant and the standards was concentration-dependent and compared favorably with both BHT and rutin. The scavenging activity was in the following order: BHT > rutin > acetone extract > aqueous extract. The acetone extract's activity was significantly higher than the two standards while aqueous extract showed significantly lower inhibitory activity (*P* < 0.05) (Figure 7). The IC_50_ values of the plant extracts/standards were in the following increasing order: acetone extract < BHT < rutin < aqueous extract (Table 1).

The results show that the free radical scavenging potential of the plant extracts varies according to free radical species. Both the acetone and aqueous extracts showed high activity for the radical scavenging potentials with acetone extract exhibiting the highest potentials in all assay models except with reducing power and nitric oxide scavenging ability.

### 3.3. *In Vitro* Anti-Inflammatory Activity

Acetone extract protected the albumin from denaturation in a dose-dependent manner which was comparable to the standard drug, except at the concentration of 300 *μ*g/mL. The aqueous extract also shows significant protein denaturation inhibition with increase in the concentration (100 to 300 *μ*g/mL) when compared to the standard drug. However, it showed a significant decrease from 400 *μ*g/mL (*P* < 0.05) (Table 2).

The acetone and aqueous extracts' protection against erythrocyte membrane lysis is significantly lower than the standard drug in a dose-dependent manner, except for the aqueous extract at 500 *μ*g/mL which was similar in inhibition (*P* < 0.05) (Tables 1 and 3). The IC_50_ values indicate that acetone extract exhibited the highest protein denaturation inhibitory effect compared to the aqueous extract while the aqueous extract exhibited the highest membrane lysis protection (Table 1).

### 3.4. Antibacterial Analysis

The species was tested against eight diabetic status opportunistic bacteria. The results show that the acetone and aqueous extracts were active against the growth of all organisms except* S. marcescens*. The zones of inhibition were varying from 12 to 41 mm (Table 4).

The highest activity against the tested bacteria was obtained with the aqueous extract which showed inhibition zones diameters of 41, 19, and 18 mm against* S. aureus*,* P. vulgaris*, and* S. flexneri*, respectively (Table 4), while with acetone extract's inhibitory activity was with inhibition zones diameters of 35, 24, 23, and 20 mm against* S. aureus*,* E. faecalis*,* S. flexneri*, and* P. vulgaris*, respectively. The lowest activity obtained with aqueous extract showed inhibition zone diameter of 12 mm against* P. aeruginosa*, while with acetone extract activity against* S. pyogenes* showed the lowest inhibition zones diameter of 14 mm (Table 4). Based on the overall mean inhibition diameters, acetone extract showed significantly more inhibitory activity with overall mean inhibition diameters of 18.95 ± 9.38 mm than aqueous extract with 17.09 ± 10.60 mm inhibition diameter (*P* < 0.05).

The varying concentrations between 5 and 0.005 mg/mL of the plant extracts were tested in order to determine their MICs (Table 5). The lowest MIC (0.31 mg/mL) with acetone extract was observed against* S. aureus*,* P. aeruginosa*, and* E. faecalis*. The aqueous extract showed the lowest MIC activity against* S. aureus* (0.078 mg/mL) and* S. flexneri* (0.31 mg/mL). The acetone extract (MIC of 1.25 mg/mL) showed significantly similar activity with the standard drug/control (Amoxicillin) against* P. vulgaris* (*P* < 0.05) (Table 5).

## 4. Discussion

The results obtained in the present study revealed the presence of considerable amounts of total phenols, flavonoids, flavanols, proanthocyanidins, and alkaloids in acetone and aqueous extracts with the latter extracting more amounts (Figure 1). The aqueous solvent extracted more of the phytochemicals indicating differences in the extracting capacity of the two solvents. The* in vitro* antioxidant model assays revealed that the plant species possesses free radical scavenging potential varying with free radical species (Figures 2–7). The antioxidant potential of this species could be postulated by the presence of these phytochemicals. Phenolic compounds and flavonoids are the major constituents in most plants reported to possess free radical scavenging activity [31]. The mode of action of phenolic compounds in free radical moping activity is via inactivating lipid free radicals or by preventing the decomposition of hydroperoxides into free radicals [32]. Flavonoids possessing hydroxyl groups mediate their antioxidant effects by chelating metal ions and quenching or protecting antioxidant defenses [32].

Proanthocyanidins are a class of nutrients that belong to the flavonoid family; hence their mechanism of action as antioxidants is the same as that of flavonoids [33]. These exert their activity via inactivating the iron ions by chelating and suppressing the superoxide-driven Fenton reaction, which is the most important source of reactive oxygen species [33].

Alkaloids have also been shown to have antioxidant properties via alleviating H_2_O_2_-induced oxidative damage [34]. Flavanols also have free radical scavenging potential through lipid peroxidation inhibition with its activity due to the sugar substitutions in the phenolic C ring [34]. Saponins possess antioxidant potential through hydrogen peroxide scavenging capability [35]. Tannins are potent antioxidants, whose bioactivities are through chelating metal ions such as Fe (II) and interfering with one of the reaction steps in the Fenton reaction and thereby retard oxidation. Tannins also inhibit lipid peroxidation via the inhibition of cyclooxygenase enzyme [35].

Protein denaturation and membrane leakage are the main cause of inflammatory processes implicated in pathogenesis of diseases and infections [3]. This plant species exhibited remarkable protein denaturation inhibitory activity while offering high protection against erythrocyte membrane lysis hence indicating anti-inflammatory potential (Tables 1, 2, and 3). This bioactivity could be postulated due to the presence of these phytochemical constituents which may be capable of inhibiting the inflammation processes [3]. Studies have shown that phenolics and flavonoids act as excellent anti-inflammatory agents. Flavonoids are reported to have anti-inflammatory activities and act through inhibiting a number of immune system inflammatory mediators [36]. Alkaloids may also inhibit inflammation through blocking the cyclooxygenase and lipoxygenase metabolic pathways of arachidonic acid metabolism [37]. Proanthocyanidins also possess anti-inflammatory and immunomodulatory properties which could help to prevent oxidative stress related diseases [29].

The acetone and aqueous extracts of* B. abyssinica* showed activity against growth of some pathogenic bacteria that cause infections associated with diabetic status (Tables 4 and 5). Both plant extracts greatly inhibited the growth of* S. aureus*,* P. vulgaris*,* S. flexneri*, and* E. faecalis* (Tables 4 and 5). The antibacterial activity could also be attributed by the presence of some chemical constituents identified in the plant fractions [38].

The total phenolics and the highest phytochemicals have been reported to have antibacterial activity [38]. Alkaloids and flavonoids also possess antibacterial activity. The antibacterial mechanisms of action of selected flavonoids are attributed to inhibition of DNA gyrase, cytoplasmic membrane function, and licochalcones A and C energy metabolism [39]. Proanthocyanidins are also known to be targets as antibacterial agents [40].

These findings also showed some trace amounts of saponins and tannins (Figure 1). Antibacterial activity of saponins from different plant sources has been widely reported [41]. Tannins act as natural antibiotics through preventing lipid peroxidation or by iron deprivation, hydrogen bonding, or specific interactions with vital proteins such as enzymes in microbial cells [41]. Tannins have been further shown to confer inhibitory growth activity against* S. aureus*,* P. aeruginosa*, and* E. faecalis* [42]. Therefore, these classes of phytochemical constituents are promising prospects for future synthesis of new antibacterial agents [42].

Even though these phytochemical constituents are known to have antioxidant, anti-inflammatory, and antibacterial properties, it is postulated that they could be acting singly or in combinations to potentiate the plants' potentials [43]. Therefore, it is crucial to isolate and elucidate the bioactive compounds and determine their pharmacological properties. This could facilitate the finding of novel antioxidant, antibacterial, and anti-inflammatory agents.

The presence of phytochemicals such as total phenolics, alkaloids, flavonoids, saponins, tannins, and proanthocyanidins in* B. abyssinica* provides some scientific evidence for the biological activities and also accounts for the multipharmacological use of this plant in traditional medicine. The plant extracts showed good antioxidant, anti-inflammatory, and antibacterial activities indicating that this plant is a good source of these agents.

## Figures and Tables

**Table 1:** Scavenging and inflammation inhibitory activities of acetone and aqueous extracts of *B. abyssinica*.

Activity	A		B		C		D		E		F		G		H	
Samples	IC_50_^a^	*R* ^2^ ^b^	IC_50_^a^	*R* ^2^ ^b^	IC_50_^a^	*R* ^2^ ^b^	IC_50_^a^	*R* ^2^ ^b^	IC_50_^a^	*R* ^2^ ^b^	IC_50_^a^	*R* ^2^ ^b^	IC_50_^a^	*R* ^2^ ^b^	IC_50_^a^	*R* ^2^ ^b^
Acetone extract	1.25	78.3	0.19	79.6	0.23	89.9	0.04	88.4	0.18	83.6	0.14	86.2	0.22	74.3	0.51	90.1
Aqueous extract	0.19	79. 7	0.26	99.7	0.14	85.9	0.24	99.8	1.61	92.2	0.21	87.8	0.33	71.5	0.33	72.2
BHT	0.18	88.7	0.2	90.4	—	—	0.25	99.4	2.04	84.8	0.16	87.3	—	—	—	—
Vitamin C	0.12	85.3	0.07	89.7	0.09	84.9	0.22	93.1	2.21	96.8	—	—	—	—	—	—
Rutin	—	—	0.26	81.4	0.23	91.1	—	—	—	—	0.18	81.3	—	—	—	—
Gallic acid	—	—	—	—	—	—	—	—	1.67	76.7	—	—	—	—	—	—
Diclofenac sodium	—	—	—	—	—	—	—	—	—	—	—	—	0.31	91.9	0.48	81.2

A is reducing power; B is DPPH scavenging activity; C is nitric oxide scavenging activity; D is hydrogen peroxide scavenging activity; E is lipid peroxide scavenging activity; F is ABTS scavenging activity; G is protein denaturation inhibitory activity; and H is membrane lysis inhibitory activity. ^a^IC_50_ is defined as the concentration (mg/mL) sufficient to obtain 50% of a maximum scavenging capacity. ^b^Coefficient of determination. Values are obtained from regression lines with 95% confidence level. — indicates values not determined.

**Table 2:** Protein denaturation activity of acetone and aqueous extracts of *B. abyssinica*.

Concentration (*μ*g/mL)	Standard (diclofenac sodium)	Acetone extract of *B. abyssinica*	Aqueous extract of *B. abyssinica*
	Mean % inhibition ± SD	Mean % inhibition ± SD	Mean % inhibition ± SD
500	98.68 ± 2.28^a^	85.09 ± 0.76^b^	67.98 ± 11.50^c^
400	95.61 ± 0.76^a^	79.39 ± 0.75^b^	78.95 ± 2.63^b^
300	87.72 ± 1.52^a^	72.81 ± 5.93^a^	85.09 ± 21.43^a^
200	88.16 ± 2.01^a^	71.06 ± 2.28^b^	93.86 ± 2.01^a^
100	83.77 ± 0.76^a^	30.70 ± 5.32^b^	86.40 ± 0.76^a^

Data expressed as means ± SD; *n* = 3; values along a row with different superscripts are significantly different (*P* < 0.05).

**Table 3:** Effect of acetone and aqueous extracts of *B. abyssinica* on erythrocyte membrane haemolysis.

Concentration (*μ*g/mL)	Standard (diclofenac sodium)	Acetone extract of *B. abyssinica*	Aqueous extract of *B. abyssinica*
	Mean % inhibition ± SD	Mean % inhibition ± SD	Mean % inhibition ± SD
1000	89.19 ± 1.78^a^	70.72 ± 0.77^b^	79.50 ± 1.41^c^
500	79.95 ± 6.14^a^	56.31 ± 0.78^b^	75 ± 1.17^a^
400	79.51 ± 2.17^a^	34.69 ± 0.79^b^	62.61 ± 2.17^c^
300	63.06 ± 2.81^a^	30.63 ± 0.39^b^	44.14 ± 0.78^c^
200	58.56 ± 1.03^a^	31.08 ± 0.68^b^	41.89 ± 1.35^c^
100	58.55 ± 0.78^a^	21.4 ± 0.78^b^	16.44 ± 0.39^c^

Data expressed as means ± SD; *n* = 3; values along a row with different superscripts are significantly different (*P* < 0.05).

**Table 4:** Inhibition zone diameters caused by acetone and aqueous extracts of *B. abyssinica* (50 mg/mL) against the tested opportunistic bacteria.

Sample	Inhibition zone diameter (mm)							
	Sf	Pa	Sa	Ef	Kp	Sp	Pv	Sm
Acetone extract	23.33 ± 3.06_1_^a^	20 ± 3.46_1_^a^	35 ± 2.65_2_^a^	24 ± 3.46_1_^a^	14.67 ± 0.58_3_^a^	14.33 ± 1.53_3_^a^	20.33 ± 0.58_1_^a^	0
Aqueous extract	17.67 ± 6.03_1_^a^	12 ± 1.73_1_^b^	41 ± 3.46_2_^b^	14.67 ± 6.35_1_^b^	16 ± 1.73_1_^a^	16.67 ± 2.08_1_^a^	18.67 ± 1.15_1_^a^	0
Control	22.67 ± 2.31_1_^a^	27 ± 3.46_1_^c^	30.67 ± 3.51_2_^c^	26.67 ± 3.79_1_^a^	23.67 ± 0.58_1_^b^	38.33 ± 1.53_3_^b^	41.67 ± 2.89_4_^b^	32.56 ± 2.44_2_

The bacteria isolates are denoted as Sf (*Shigella flexneri*), Pa (*Pseudomonas aeruginosa*), Sa (*Staphylococcus aureus*), Ef (*Enterococcus faecalis*), Kp (*Klebsiella pneumonia*), Sp (*Streptococcus pyogenes*), Pv (*Proteus vulgaris*), and Sm (*Serratia marcescens*). Data expressed as means ± SD; *n* = 3; values along a row with different subscripts are significantly different (*P* < 0.05). Mean with the different superscript in the same column is significantly different (*P* < 0.05). Concentration of positive control is 0.013 mg/mL.

**Table 5:** Minimum inhibitory concentrations (MIC) of acetone and aqueous extracts of *B. abyssinica* against the tested opportunistic bacteria.

Sample	MIC (mg/mL)							
	Sf	Pa	Sa	Ef	Kp	Sp	Pv	Sm
Acetone extract	0.63_1_^a^	0.31_2_^a^	0.31_2_^a^	0.31_2_^a^	2.5_3_^a^	>5_4_^a^	1.25_5_^a^	—
Aqueous extract	0.31_1_^b^	>5_2_^b^	0.078_3_^b^	1.25_4_^b^	2.5_5_^a^	5_2_^a^	0.63_6_^b^	—
Control	0.16_1_^c^	0.16_1_^c^	0.16_1_^c^	0.16_1_^c^	0.16_1_^b^	0.16_1_^b^	1.25_2_^a^	0.16_1_

The bacteria isolates are denoted as Sf (*Shigella flexneri*), Pa (*Pseudomonas aeruginosa*), Sa (*Staphylococcus aureus*), Ef (*Enterococcus faecalis*), Kp (*Klebsiella pneumonia*), Sp (*Streptococcus pyogenes*), Pv (*Proteus vulgaris*), and Sm (*Serratia marcescens*). “—” denotes values not determined. Data expressed as means ± SD; *n* = 3; values along a row with different subscripts are significantly different (*P* < 0.05). Mean with the different superscript in the same column is significantly different (*P* < 0.05). Concentration of positive control is 0.013 mg/mL.
